# Does total hip arthroplasty benefit patients with minimal radiological osteoarthritis?

**DOI:** 10.1302/2633-1462.63.BJO-2024-0103.R1

**Published:** 2025-03-12

**Authors:** Kartik Logishetty, Jeroen C. F. Verhaegen, Shannon Tse, Tim Maheswaran, Max Fornasiero, Hariharan Subbiah Ponniah, Jonathan B. Hutt, Johan D. Witt

**Affiliations:** 1 University College London Hospital, London, UK; 2 The MSk Lab, Department of Surgery and Cancer, Imperial College, London, UK; 3 University Hospital Antwerp, Antwerp, Belgium

**Keywords:** Total hip arthroplasty, Early osteoarthritis, Hip, CT, osteoarthritis (OA), total hip arthroplasty (THA), hips, Comorbidities, Oxford Hip Score (OHS), patient-reported outcome measures (PROMs), joint space narrowing, visual analogue scale, MRI scans, hypermobility

## Abstract

**Aims:**

The effectiveness of total hip arthroplasty (THA) for patients with no or minimal radiological signs of osteoarthritis (OA) is unclear. In this study, we aimed to: 1) assess the outcome of such patients; 2) identify patient comorbidities and CT or MRI findings which predicted outcome; and 3) compare their outcome to the expected outcome of THA for hip OA.

**Methods:**

Adult patients undergoing THA for hip pain, with no or minimal radiological features of OA (Tönnis grading scale ≤ 1), were identified from a consecutive series of 1,925 THAs. Exclusion criteria were: inflammatory arthritis; osteonecrosis of the femoral head; prior trauma or infection; and patients without minimum one-year follow-up and patient-reported outcome measures (PROMs). The primary outcome measure was the Oxford Hip Score (OHS). Secondary outcome measures were EuroQol-visual analogue scale (EQ-VAS), University of California and Los Angeles (UCLA) scale, and patient satisfaction on a validated three-point ‘better’, ‘same’, or ‘worse’ scale.

**Results:**

A total of 107 patients with a median age of 41 years (IQR 18 to 73) were included, with mean follow-up of 6.0 years (SD 3.1). All patients underwent a diagnostic hip injection as a decision aid. Median postoperative OHS was 34 (IQR 28 to 42), with 36 patients (33%) achieving a patient-acceptable symptom state (OHS ≥ 42), lower than THA patients in international registries (40 to 43 points). Secondary outcomes were UCLA of 6 (4 to 8) and EQ-VAS of 73 (51 to 80); 91/102 patients (89%) felt ‘better’ and would ‘undergo surgery again'. Patients with chronic pain syndrome or hypermobility had lower OHS than patients without comorbidities (-6 points, p < 0.006). Overall, 84 patients had a CT and 34 patients an MRI. Patients with subchondral cysts (OHS 42 (37 to 45) vs 35 (26 to 36); p = 0.014) or joint space narrowing on CT (OHS 42 (IQR 37 to 44) vs 35 (26 to 36); p = 0.022) had higher function.

**Conclusion:**

Despite high satisfaction levels, patients undergoing THA with minimal or no radiological OA had lower postoperative function than typical THA patients. We recommend obtaining low-dose CT imaging and a diagnostic hip injection to aid decision-making.

Cite this article: *Bone Jt Open* 2025;6(3):328–335.

## Introduction

Pain not effectively controlled by conservative methods remains the main indication for surgical intervention to the hip joint. Several hip preservation techniques are effective in improving symptoms for specific hip pathologies, potentially delaying the need for total hip arthroplasty (THA).^1,2^ There are instances, however, where patients are not ideally suited to hip preservation surgery and yet have severe symptoms with few radiological signs of arthritis. Many of these patients may be relatively young when compared with the usual age of patients undergoing THA. Deciding on the best solution for these patients is difficult, because there is some evidence that THA in the absence of significant radiological signs of arthritis is not associated with a predictable outcome.^3^ In this setting, THA has been associated with not achieving minimum clinically important differences, persistent pain, and dissatisfaction after surgery. However, studies are limited either by lacking analysis of multiplanar imaging,^4,5^ or the impact of patient comorbidities on outcomes.^4,6^

The weightbearing anteroposterior (AP) pelvis radiograph is a cornerstone of diagnosing hip osteoarthritis (OA). Alongside groin pain, and painful or reduced hip internal rotation, radiological signs guide subsequent management. However, there is poor concordance between radiological signs and clinical suspicion of hip OA.^7^ Multiplanar imaging, including CT or MRI, improves diagnostic accuracy in earlier disease,^8^ and is more sensitive than radiographs in detecting specific features of OA.^9^ These methods can identify occult subchondral cysts, subtle osteophytes, labral degeneration, and posterior-inferior arthritis. They may also elucidate alternative aetiologies for hip pain, including hip dysplasia, femoroacetabular impingement, and labral tears, where hip preservation surgery could be indicated.^8^ However, among patients with early degenerative changes, hip preservation surgery is associated with less favourable outcome,^10^ while THA could be a valuable alternative.

The aim of this study was to: 1) assess the outcome of THA in hip patients with no or minimal plain radiological signs of OA; 2) identify whether patient comorbidities or multiplanar imaging findings are predictive of outcome; and 3) compare the outcome in these patients to the expected outcome of THA in hip OA as published in the literature.

## Methods

### Study design

Following institutional board review approval, a single-centre retrospective observational study was conducted of consecutive THAs performed between January 2006 and December 2021 by two surgeons (JDW, JBH) at the Young Adult Hip Unit, University College London Hospital – an academic tertiary referral centre for patients with young adult hip disease. Demographic detail was extracted from the institutional electronic health record, and patient-reported outcome measures (PROMs) were collected over a six-month period (January to July 2023), either when patients attended for routine follow-up or completed one postal questionnaire, or responded to up to three attempts at phone contact. Inclusion criteria were patients aged over 18 years who had undergone THA for hip pain, and had no or minimal radiological features of OA (Tönnis grading scale ≤ 1),^3^ and had at least one-year postoperative clinical follow-up. Exclusion criteria were prior hip trauma or infection, osteonecrosis of the femoral head, rheumatoid arthritis, juvenile idiopathic arthritis (JIA), oncological hip disease, Tönnis grade^3^ ≥ 2, or those for whom postoperative patient-reported outcome measures (PROMs) were not available.

Demographics collated were sex, age, the primary hip pathology, comorbidities, and incidence of further surgery to the operated hip, including revision. Hypermobility was determined if patients had a pre-existing diagnosis of a hypermobility syndrome or had a Beighton score^11^ ≥ 6 on examination. From the institutional database of 1,925 THAs, 217 hips met the inclusion criteria. Of these, 104 hips did not have PROMs available and were excluded; 107 hips (102 patients, 90 F:12 M, median age 40.6 years (IQR 35.1 to 45.8; range 18 to 73)) were included for analysis. Preoperative weightbearing anteroposterior and lateral plain radiographs were evaluated using the Tönnis grading scale (Table I) by two independent raters (KL, JDW). It consists of three progressive degrees of degenerative changes to the hip joint, accounting for sclerosis and joint space narrowing noted in Grade 1, before the progressive development of cysts, femoral head asphericity, and advanced joint space narrowing in Grade 2 and 3. CT and MRI scans were assessed by radiologists specializing in musculoskeletal disease. MRI reports were interrogated for the presence of chondral damage, subchondral cysts, or labral injury; and CT reports for the presence of joint-space narrowing, subchondral cysts, osteochondral defects, and osteophytes.

**Table I.** Tönnis grading scale of hip arthritis.

Grade	Radiological features
0	No signs of osteoarthritis
1	Slight narrowing of joint space
	Slight lipping at joint margin
	Slight sclerosis of the femoral head or acetabulum
2	Small cysts in femoral head or acetabulum
	Increasing joint space narrowing
	Moderate loss of femoral head sphericity
3	Large cysts
	Severe joint space narrowing
	Severe deformity of the femoral head
	Avascular necrosis

### Outcome measures

The primary outcome measure was hip function, as assessed by the Oxford Hip Score (OHS).^12^ We collected data for four secondary outcomes: further surgery to the operated hip, including revision of implants; EuroQol-visual analogue scale (EQ-VAS);^13^ the University of California Los Angeles (UCLA) activity scale;^14^ the International Hip Outcome Tool-12 (iHOT-12);^15^ and a patient satisfaction question previously used to validate PROMs after THA.^16^ Patients were asked, ‘Knowing what you know now, and related to how your hip feels today, would you say 1) My hip feels better (B), and I would have the surgery again; 2) my hip is about the same (S), and I am unsure whether I would have the surgery again; or 3) my hip feels worse (W), and I would not have the surgery again’? This was recorded as a B/S/W state.

### Surgical technique

A diagnostic intra-articular hip injection was performed using 5 ml 0.5% Marcaine under fluoroscopic or ultrasound guidance, to confirm that pain was arising from the hip joint itself, and not neurogenic or arising from surrounding soft-tissues. A positive response was used as a decision aid for whether THA would improve symptoms. Patients were referred for hip-specific physiotherapy, and weight-loss management where BMI exceeded 35 kg/m^2^, although BMI was not a contraindication to surgery. Those who failed conservative management were offered THA. Cementless implants were used in 106/107 hips (99%), of which 94/107 (87.8%) were a Furlong Evolution or Furlong H.A.C stem (JRI, UK) with a CSF acetabular component (JRI). THA was performed using either the direct anterior (70/107, 65.4%) or posterior surgical approach, based on surgeon preference. All but one THA (106/107, 99%) had ceramic-on-ceramic bearings.

### Statistical analysis

Statistical analyses were performed using RStudio software v 4.3.0 (Posit, USA). The Kolmogorov-Smirnov test showed that data for the primary outcome measure – OHS – were not normally distributed. The Kruskal-Wallis test was used to determine if there were differences between continuous PROMs for the primary diagnosis or comorbidities, and Wilcoxon rank-sum test for post-hoc pairwise comparisons. Fisher’s exact test was used to detect differences between the primary hip diagnoses, and chi-squared test for the categorical PROM – B/S/W. Odds ratios with 95% CIs were calculated to determine the effect size when there were differences between diagnoses or B/S/W state. Mann-Whitney U test was used to compare whether the presence or absence of CT and MRI findings was associated with PROMs. Linear regression was used to detect associations between outcome and independent variables. A type 1 error rate of 5% (p < 0.05) was accepted to detect a statistically significant difference.

## Results

In total, 107 hips (102 patients, median age 40.6 years; range 18 to 73; IQR 35.0 to 48.0) were available for analysis with a mean follow-up of 6.0 years (SD 3.1). The most common primary hip diagnosis was hip dysplasia (55 patients (49.5%) Table II), and most patients (72.0%) had no comorbidities (Table III). All patients had a hip injection, 24 hips (22.4%, 18 F:6 M) had undergone prior hip arthroscopy, and four hips (3.7%, 4 F:0 M) had prior periacetabular osteotomy (PAO). At latest follow-up, one hip (0.9%) had undergone revision surgery – for femoral implant fixation failure within one year of surgery. There were no periprosthetic fractures, dislocations, or periprosthetic joint infections. A total of 20 hips (18.7%; 19 patients, 14 F:5 M) had further interventions: an image-guided corticosteroid injection for greater trochanteric pain syndrome (3/107 hips; 2.8%), psoas tendinopathy (15/107; 14.0%), or lateral femoral cutaneous nerve neuropathy (1/107; 0.9%); and repair of a fascial dehiscence (1/107; 0.9%).

**Table II.** Primary hip diagnosis of patients undergoing total hip arthroplasty.

Primary hip diagnosis	Hips, n (%)
Hip dysplasia	53 (49.5)
Pincer-type FAI	18 (16.8)
Acetabular retroversion	13 (12.1)
Cam-type FAI	6 (5.6)
SCFE or Perthes' disease	5 (4.7)
Persistent/recurrent pain post-PAO	4 (3.7)
Primary osteoarthritis	4 (3.7)
Skeletal dysplasia	2 (1.8)
Synovial chondromatosis	2 (1.8)

FAI, femoroacetabular impingement; PAO, periacetabular osteotomy; SCFE, slipped capital femoral epiphysis.

**Table III.** Comorbidities of patients undergoing total hip arthroplasty.

Comorbidities	Hips, n (%)
None	77 (72.0)
Chronic pain*	10 (9.3)
Hypermobility	15 (14.0)
Spinal disease†	8 (7.5)
Neurological or neuromuscular disease‡	4 (3.7)
Autoimmune disease§	3 (2.8)

* Chronic pain, diagnosed using International Association for the Study of Pain criteria, by a specialist in pain management.^17^

† Spinal disease included patients with lower back pain, scoliosis, or lumbar stenosis.

‡ Neurological or neuromuscular disease included patients with cerebral palsy, polio, or multiple sclerosis.

§ Autoimmune disease included patients with Sjögren's disease, hypothyroidism, Crohn’s disease, systemic lupus erythematosus, and antiphospholipid syndrome.

### The influence of primary hip diagnosis on outcomes after THA

**Fig. 1:**
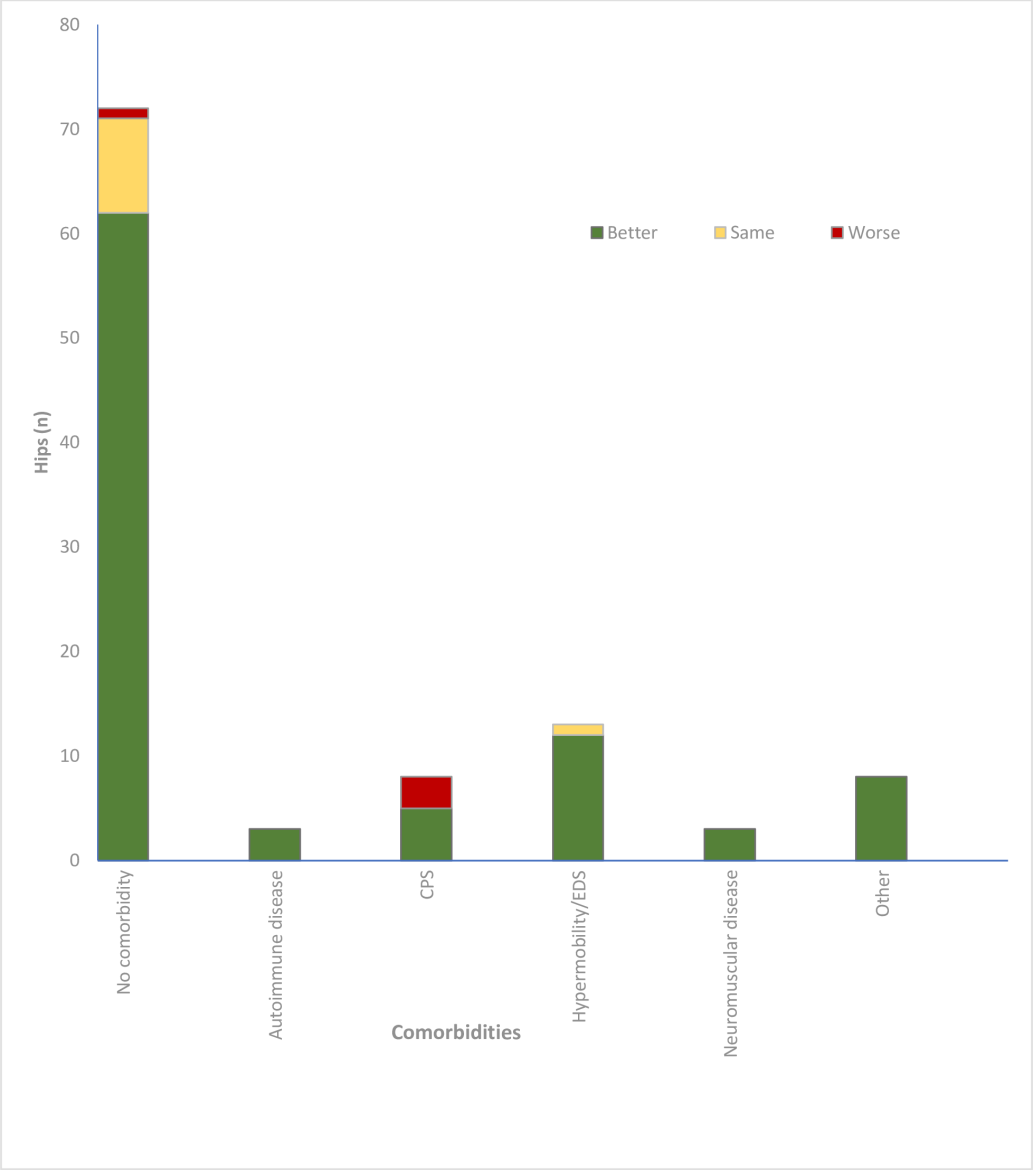
The impact of comorbidity on patient satisfaction and willingness to undergo surgery again. CPS, chronic pain syndrome; EDS, Ehlers-Danlos syndrome.

The median postoperative OHS for patients was 34.3 (13.9, IQR 13 to 48), and 36/107 hips (33.6%) achieved an OHS ≥ 42, a previously validated patient-acceptable symptom state (PASS).^18^ Other PROMs are reported in Table IV. There was no association between a patient’s primary hip diagnosis and their postoperative PROMs (p = 0.660, Kruskal Wallis test). A total of 91 of the 102 patients (93 hips; 89.2%) reported that their hip pain and function was Better than prior to THA and they would have the surgery again, seven patients (ten hips; 6.8%) felt the same, and four patients (four hips; 3.9%) felt worse and would not have the surgery again (Figure 1). There was no association between a patient’s primary hip diagnosis and whether they reported the operated hip function was B/S/W than prior to THA (p = 0.119, chi-squared test).

**Table IV.** Patient-reported outcome measures.

PROM	Median	Range	IQR
OHS (n = 103)	34.3	13 to 48	28.2 to 42.2
UCLA (n = 107)	5.6	3 to 10	4.4 to 8.0
iHOT-12 (n = 102)	78.0	9 to 100	50.6 to 91.9
EQ-VAS (n = 101)	72.5	20 to 99	50.5 to 80.3

EQ-VAS, EuroQol-visual analogue scale; iHOT-12, International Hip Outcome Tool-12; OHS, Oxford Hip Score; PROM, patient-reported outcome measure; UCLA, University of California Los Angeles activity scale.

### The influence of comorbidities on outcomes after THA

Patients with chronic pain – diagnosed in a specialist pain clinic using the International Association for the Study of Pain criteria^19^ – had significantly lower OHS, iHOT12, and UCLA scores (p < 0.05, Wilcoxon rank-sum test; Table V); and were more likely to report being Worse after THA (odds ratio (OR) 2.2, 95% CI 2.0 to 2.4; p < 0.001, Wilcoxon rank-sum test) compared to those with no comorbidities. Three of the four patients in this cohort who reported being worse had chronic pain.

**Table V.** The impact of comorbidity on patient-reported outcome measures.

Comorbidity	Estimates	95% CI	p-value*
**OHS (n = 103)**			
Chronic pain	-5.1	-11.8 to -3.6	0.037
Hypermobility	-5.7	-11.1 to -0.3	0.039
Spinal disease	-2.1	-8.8 to 4.6	0.537
Neurological or neuromuscular disease	5.6	-5.1 to 16.2	0.301
Autoimmune disease	-3.4	-14.1 to 7.2	0.523
**UCLA Activity Scale (n = 107)**			
**Comorbidity**			
Chronic pain	-2.6	-4.2 to -1.0	0.001
Hypermobility	-1.0	-2.3 to 0.3	0.134
Spinal disease	-1.6	-3.2 to 0.0	0.045
Neurological or neuromuscular disease	1.2	-1.3 to 3.7	0.350
Autoimmune disease	-0.8	-3.3 to 1.7	0.530
**iHOT12 (n = 102)**			
**Comorbidity**			
Chronic pain	-23.1	-41.4 to -4.8	0.014
Hypermobility	-3.9	-18.2 to 10.5	0.595
Spinal disease	-12.0	-30.3 to 6.3	0.196
Neurological or neuromuscular disease	12.5	-16.5 to 41.4	0.395
Autoimmune disease	-12.1	-41.0 to 16.8	0.409
**EQ-VAS (n = 101)**			
**Comorbidity**			
Chronic pain	4.7	-10.5 to 19.9	0.541
Hypermobility	-20.6	-32.9 to -8.3	0.001
Spinal disease	-14.4	-29.6 to 0.8	0.063
Neurological or neuromuscular disease	-12.6	-36.6 to 11.5	0.303
Autoimmune disease	-22.9	-46.9 to 1.2	0.062

* Wilcoxon rank-sum test.

EQ-VAS, EuroQol-visual analogue scale; iHOT-12, International Hip Outcome Tool-12; OHS, Oxford Hip Score; UCLA, University of California Los Angeles activity scale.

Linear regression showed that when compared to having no comorbidities, only chronic pain was significantly associated with an average decrease of 5.1 points on the OHS, 2.6 points on the UCLA scale (p = 0.001), and an average decrease of 23.1 points on the iHOT-12 score compared to no comorbidities (p = 0.014; Table V).

Having hypermobility (including Ehlers-Danlos syndrome (EDS)) was associated with an average decrease of 5.7 points on the OHS compared to no comorbidities (p = 0.039; Table V). Hypermobility was also significantly associated with an average decrease of 20.6 points on the EQ-VAS scale compared to no comorbidities (Table V).

### Influence of MRI and CT abnormalities on outcomes after THA

In total, 34/107 hips had undergone an MRI scan prior to THA; 16/34 hips (47.1%) reported labral tears, and 17/34 (50.0%) hips had chondral abnormalities. The presence of a labral tear was not associated with a difference in PROMs or B/S/W state after THA. Those with chondral abnormalities had significantly higher postoperative EQ-VAS scores than those without (mean 68.2 (SD 19.1) vs 53.8 (18.3); p = 0.042), and no difference in other PROMs or B/S/W state.

In total, 84/107 hips had undergone a CT scan prior to THA; 42/84 hips (50.0%) had subchondral cysts, and 43/84 hips (51.2%) had joint space narrowing. The presence of cysts was associated with a significantly higher postoperative OHS (median 42.4 (IQR 37.0 to 44.8) vs 34.6 (IQR 25.6 to 36.2); p = 0.014, Mann-Whitney U test), and no difference in other PROMs. Patients with hips with cysts were significantly more likely to report their hip feeling ‘better’ after THA compared to those without cysts (OR 1.3, 95% CI 1.2 to 1.5; p = 0.011, chi-squared test). Those with joint space narrowing had significantly higher postoperative OHS (median 41.6 (IQR 37.2 to 43.6) vs 35.0 (IQR 25.9 to 36.3); p = 0.022, chi-squared test), iHOT-12 (EQ-VAS scores (mean 5.9 (SD 2.2) vs 5.3 (SD 2.0); p = 0.021, chi-squared test)), and were more likely to report their hip feeling ‘better’ after THA than those without joint space narrowing (OR 1.2, 95% CI 1.1 to 1.4; p = 0.023, chi-squared test).

## Discussion

Young hip patients with no or minimal radiological OA can expect significant improvement following THA, with almost 90% reporting to feel ‘better’ after surgery and, knowing what they know now, would have their operation again. However, only one in three patients achieved a PASS when the OHS threshold was ≥ 42.^18^ Presence of chronic pain and hypermobility negatively influenced outcome, whereas patients with subchondral cysts on preoperative CT scans were 30% more likely to have an improved hip function at final follow-up after THA.

### Clinical outcomes

The OHS was lower in this cohort than patients undergoing THA in registry studies. Postoperative OHS in the UKNJR,^17^ AOANJRR,^20^ and NZJR^21^ are 43.0 (IQR 36 to 47), 40.4 (SD 7.6), and 41.5 (SD 7.5), respectively. The median EQ-VAS reported in this study (70.3, IQR 50 to 80) was also lower than in the UKNJR (80, IQR 70 to 90) and the AOANJRR (81.3, SD 15.6). It is important to note that patients in this study are atypical: they were predominantly female (88%), and younger (mean 40.6 years (SD 11.0)) than those undergoing THA in national registries, or in studies reporting THA in comparable patients with available PROMs.^22,23^ Patients with rheumatoid arthritis, JIA**,** or femoral head osteonecrosis were excluded, so direct comparisons of PROMs to studies of younger patients undergoing THA for all indications are of limited value.

A proximate study by Sharrock et al^23^ reported that only 16/70 patients (23%) (mean age 60 years, 61% female) with early radiological OA had a ‘successful THA’, defined as achieving OHS ≥ 42, and their average EQ-VAS was 66 points. The authors did not report how many patients had alternative pathologies which may have explained their hip pain, or the role of a diagnostic hip injection as a decision aid.^23^ In the present study, all patients had a positive response to hip injection; 36/107 (34%) hips achieved the PASS, although there was a higher EQ-VAS (median 72.5). This is similar to UK, Sweden, New Zealand, and Australian registry studies showing that 85% to 92% of all patients undergoing THA felt ‘significantly better’, were ‘satisfied’ with surgery, or achieved a minimal clinically important difference (MCID) in the OHS.^17,20,24^ The high proportion (89%) of patients in our study who reported that their hip pain and function is better and that they would have the surgery again, further highlights the challenge in identifying PROM thresholds which are meaningful to all patients undergoing THA.

### The effect of comorbidities on outcomes

Preoperative chronic pain is associated with heightened postoperative pain sensitivity,^25^ and these patients had significantly lower PROMs than those with no comorbidities and were over twice as likely to feel the same, or worse, after THA. We recommend specialist referral and conservative measures before considering THA. Patients with hypermobility reported lower OHS and EQ-VAS, but equivalent B/S/W state, as those without hypermobility. The existing literature reports similar PROMs but higher dislocation and revision rates in patients with EDS.^26^

### Effect of CT and MRI findings on outcomes

In the present study, all patients had a CT and/or MRI prior to THA – either prior to referral to the young adult hip unit or as part of subsequent investigations for their hip pain. CT imaging detected subchondral cysts or joint space narrowing in half of patients, which were not noted on plain pelvis radiographs. Compared to those without cysts, patients with cysts had significantly higher postoperative OHS (42 vs 35 points; p = 0.014), and were 30% more likely to report that their hip felt better after THA. Similarly, joint space narrowing was associated with higher OHS and EQ-VAS, and patients were 20% more likely to report that their hip felt better and they would undergo the surgery again. These findings are in agreement with Sharrock et al,^23^ who found that the presence of joint space narrowing on CT was associated with a successful THA; they also found that patients with cysts reported higher postoperative OHS than those without (36 vs 26 points; p = 0.019). The presence of chondral damage on MRI was associated with a higher postoperative EQ-VAS alone, but there is a paucity of comparable literature. CT is widely accessible and our current preferred modality for assessing hip joint morphology, degenerative changes, and lower limb rotational profile novel; however, MRI sequences which adequately visualize bone architecture may provide similar diagnostic information.^27^

### The role of a diagnostic hip injection

A hip injection is a useful decision aid in such patients.^23^ It is highly predictive of pain relief after THA,^28,29^ and is effective in differentiating the source of atypical hip pain.^29^ However, we posit that a hip joint injection may be poorly specific to diagnosing pain arising from the articular surface in patients with hypermobility, and thus laxity in the surrounding capsuloligamentous anatomy.^30^

### Decision-making for patients with hip pain and minimal or no radiological OA

The decision to offer THA in a young patient cohort is challenging, more so in the absence of significant radiological arthritis. Even early degenerative changes can negatively influence outcome of hip preservation surgery,^31,32^ and increase likelihood of progression to THA.^10,33^ These patients therefore pose a particular challenge to hip surgeons. THA instead of hip preservation surgery or delaying arthroplasty can provide immediate and long-term improvement in quality of life and function; the longevity of modern implants makes THA highly cost-effective.^34^ While a comparison between different surgical strategies was outside the scope of this study, the results support the selective use of THA in this cohort.

This study has several limitations. Patients presented to a specialist young adult hip unit, and the results may not be generalizable**,** while 50% of eligible patients did not have available PROMs and were excluded from analysis. Second, we did not measure PROMs preoperatively, which did not allow us to assess the improvement of patients and whether patients surpassed MCID thresholds – the PASS and B/S/W state are proxies. Third, the six-year follow-up period may be considered short for this cohort of young patients. However, the revision rate is low (< 1%), and the longer-term survivorship of uncemented implants with ceramic bearings is excellent.^35^ Further, the standard deviation of follow-up was over three years and it is recognized that THA PROMs decline over time.^36^ Fourth, less than one-third of patients had comorbidities, or had an MRI scan. The lack of association between most comorbidities or MRI findings, to the postoperative OHS may be due to Type 2 error. In particular, there were few patients with spinal disease, neurological disease, or autoimmune disease in this cohort; larger groups are required to make meaningful comparisons, even in the presence of statistically significant findings in this study. Last, we have used the Tönnis grading scale as it is specific to the hip joint.^3^ It has been criticised as its interobserver reliability, when differentiating between no arthritis (Grade 0) and early arthritis (Grade 1), is poor.^37^ However, in this study, it was used to differentiate between those with early and advanced hip degeneration, so its utility as a screening tool remains.

In conclusion, younger patients undergoing THA with no or minimal radiological OA had lower postoperative OHS than the general population undergoing THA, although most felt symptomatically better and knowing what they know now, they would have surgery again. Those with chronic pain syndrome or hypermobility were less likely to benefit. Patients with subchondral cysts or joint space narrowing on CT imaging were more likely to achieve higher functional scores and satisfaction. Deciding to offer THA in patients without significant radiological arthritis is complex.^38^ We recommend obtaining CT imaging with established low-dose protocols for hip surgery,^39,40^ and a diagnostic hip joint injection to aid in shared decision-making. Prospective trials are required to compare the effectiveness of THA over hip preservation in the presence of early degenerative changes.

**Take home message**

- Younger patients with minimal radiological osteoarthritis undergoing total hip arthroplasty (THA) achieve lower postoperative Oxford Hip Scores, but generally experience significant and clinically meaningful symptomatic improvement and would choose surgery again.

- Patients with chronic pain syndrome or hypermobility are less likely to benefit, while those with subchondral cysts or joint space narrowing on CT are more likely to achieve better functional outcomes and satisfaction.

- Decision-making for THA in patients without significant radiological arthritis is complex; CT imaging with low-dose protocols and diagnostic hip joint injections are recommended to guide patient selection.

## Data Availability

The datasets generated and analyzed in the current study are not publicly available due to data protection regulations. Access to data is limited to the researchers who have obtained permission for data processing. Further inquiries can be made to the corresponding author.
